# Rapid CO_2_ changes cause oscillations in photosynthesis that implicate PSI acceptor-side limitations

**DOI:** 10.1093/jxb/erad084

**Published:** 2023-03-08

**Authors:** Alan M McClain, Thomas D Sharkey

**Affiliations:** Michigan State University-Department of Energy Plant Research Laboratory, East Lansing, MI 48824, USA; Department of Biochemistry and Molecular Biology, Michigan State University, East Lansing, MI 48824, USA; Plant Biotechnology for Health and Sustainability, Michigan State University, East Lansing, MI 48824, USA; Michigan State University-Department of Energy Plant Research Laboratory, East Lansing, MI 48824, USA; Department of Biochemistry and Molecular Biology, Michigan State University, East Lansing, MI 48824, USA; Plant Resilience Institute, Michigan State University, East Lansing. MI 48824, USA; University of Essex, UK

**Keywords:** Acceptor side limitation, ATP synthase, dynamic assimilation technique, elevated CO_2_, oscillation, photosynthesis, triose phosphate utilization

## Abstract

Oscillations in CO_2_ assimilation rate and associated fluorescence parameters have been observed alongside the triose phosphate utilization (TPU) limitation of photosynthesis for nearly 50 years. However, the mechanics of these oscillations are poorly understood. Here we utilize the recently developed dynamic assimilation techniques (DATs) for measuring the rate of CO_2_ assimilation to increase our understanding of what physiological condition is required to cause oscillations. We found that TPU-limiting conditions alone were insufficient, and that plants must enter TPU limitation quickly to cause oscillations. We found that ramps of CO_2_ caused oscillations proportional in strength to the speed of the ramp, and that ramps induce oscillations with worse outcomes than oscillations induced by step change of CO_2_ concentration. An initial overshoot is caused by a temporary excess of available phosphate. During the overshoot, the plant outperforms steady-state TPU and ribulose 1,5-bisphosphate regeneration limitations of photosynthesis, but cannot exceed the rubisco limitation. We performed additional optical measurements which support the role of PSI reduction and oscillations in availability of NADP^+^ and ATP in supporting oscillations.

## Teaser

Photosynthetic CO_2_uptake at high rates sometimes oscillates, especially when CO_2_changes rapidly. We present evidence that this results from unbalanced interactions between electron transport metabolism and carbon metabolism.

## Introduction

The triose phosphate utilization (TPU) limit on the photosynthetic rate can appear when plants are capable of producing phosphorylated Calvin–Benson cycle intermediates faster than these intermediates can be dephosphorylated and converted to end-products (Sharkey, 1985a). When TPU limited, inorganic phosphate is not released from the organic phosphate pool fast enough to sustain maximum throughput of both the ATP synthase and the Calvin–Benson cycle, and photosynthesis must be down-regulated to balance the two. This regulation imposes a cap on the rate of CO_2_ fixation at the rate of end-product synthesis. Plants are not typically TPU limited under ambient conditions (Sage and Sharkey, 1987; Ellsworth *et al.*, 2015), and TPU limitation is most easily seen by elevating the rate of photosynthesis through increased light level and CO_2_ partial pressure or decreased O_2_ partial pressure (Sharkey *et al.*, 1986b) such that the photosynthetic rate is increased by 10% or 20% relative to ambient conditions (Yang *et al.*, 2016). It is more likely to be observed when photosynthesis is measured at a lower temperature than growth conditions (Stitt, 1986; Sage and Sharkey, 1987; Labate and Leegood, 1988), due to the high temperature sensitivity of end-product synthesis (Stitt and Grosse, 1988; Leegood and Edwards, 1996), which exceeds the temperature sensitivity of the other biochemical processes in photosynthesis (Cen and Sage, 2005; Sage and Kubien, 2007). The occurrence of TPU limitation depends greatly on the species and the acclimation of the plant. For example, plants grown at low temperature are often resistant to TPU limitation because they develop additional sucrose-phosphate synthase activity (Cornic and Louason, 1980; Guy *et al.*, 1992; Holaday *et al.*, 1992). Expressing *Zea mays* sucrose-phosphate synthase in tomato significantly reduced the temperature at which TPU limitation was evident (Laporte *et al.*, 2001).

TPU limitation is associated with a variety of regulatory processes. TPU-limited plants exhibit a reduced rubisco activation state in as little as 1 min after imposing TPU conditions (Sharkey *et al.*, 1986a). rubisco deactivation can restore the balance between the capacities to fix carbon and convert the fixed carbon to end-products. TPU-limited plants also develop an elevated transthylakoid proton motive force (PMF) and an associated increase in energy-dependent quenching (Takizawa *et al.*, 2008; Kiirats *et al.*, 2009). This increase is probably associated with declining phosphate concentration in the stroma (Sharkey and Vanderveer, 1989) driving up the ∆G_ATP_ of the stromal ATP synthase reaction. One consequence of this regulatory arrangement is the reduction of φ_II_ as [CO_2_] increases (Sharkey *et al.*, 1988; Stitt and Grosse, 1988). The requirement for electron transport is set by the rate of photosynthesis and photorespiration. Increasing [CO_2_] reduces the rate of photorespiration under TPU-limited conditions but this does not result in an increase photosynthetic rate. Instead, φ_PSII_ will decline and then balance the rate of electron transport with the reduced requirements for electrons because of the reduced rate of photorespiration.

In TPU-limited photosynthesis, the photosynthetic rate is defined by regulatory features. To detect TPU limitation in gas exchange data, it is easiest to determine the presence of regulatory mechanisms, such as the increase in non-photochemical quenching or the decline in φ_II_ upon increasing CO_2_ (McClain and Sharkey, 2019). The CO_2_ assimilation rate (*A*) also becomes insensitive to CO_2_ or O_2_, which demonstrates that *A* is not defined by rubisco properties under TPU limitation (Sharkey, 1985b). These regulatory mechanisms can have different time constants. For example, Sharkey *et al.* (1986b) observed depletions of ATP and ribulose 1,5-bisphosphate (RuBP) and reductions in the ATP/ADP ratio and rubisco activation state 1 min after imposing TPU limitation. However, after 18 min, RuBP was higher than before imposing TPU conditions, and the ATP/ADP ratio and rubisco activation recovered partially. Thus, as different regulatory mechanisms are induced upon imposition of TPU limitation, there can be transients in the specific process setting the rate of photosynthesis, for example the availability of RuBP at one time versus the activation of rubisco at another time.

One consequence of these transients is oscillations in *A*, which have been observed under TPU limitation (Ogawa, 1982; Walker *et al.*, 1983; Sivak and Walker, 1986, 1987). Oscillations are commonly seen when the environmental conditions are rapidly changed to elevate the photosynthetic rate, such as a step change in CO_2_ partial pressure or light availability, or a reduction in O_2_ partial pressure (Harris *et al.*, 1983) to increase carbon fixation by reducing photorespiration. Oscillations are visible in both carbon assimilation and fluorescence parameters, demonstrating parallel changes in the Calvin–Benson cycle and electron transport (Walker *et al.*, 1983; Peterson *et al.*, 1988; Stitt and Grosse, 1988). There have been a few models proposed to explain oscillations in photosynthetic rate. In general biological oscillatory models, oscillations are typically caused by a delay in a feedback component of a multiple component system, leading to overshooting of steady state before inhibition can be achieved. One theory is that there is a delay in activation of sucrose synthesis after a photosynthetic increase (Laisk and Walker, 1986). Another theory is that the delay originates from fructose-2,6-bisphosphate inhibiting fructose-1,6-bisphosphatase (Stitt *et al.*, 1984; Laisk and Eichelmann, 1989; Laisk *et al.*, 1989). One of the limiting factors in research on oscillations is that they are inherently incompatible with gas exchange measurements. Normally we would like to use gas exchange to establish the limiting processes of photosynthesis by determining how *A* varies with intercellular CO_2_ (*C*_i_) but getting a clear *A*/*C*_i_ plot during oscillations is complicated by their speed and unpredictability.

The use of ramps of CO_2_ to induce oscillations should allow us to study the phenomenology of oscillations with high-speed measurements of *A* and *C*_i_ (Stinziano *et al.*, 2017). However, the 100 ppm min^–1^ limit on ramp speed with the rapid *A*/*C*_i_ response (RACiR; Stinziano *et al.*, 2019) technique combined with inaccurate *C*_i_ measurements, especially at the beginning and end of curves, limited this approach. Dynamic assimilation techniques (DATs; Saathoff and Welles, 2021) represent a natural evolution of RACiR that features a greater range of ramp rates and better accuracy, especially at the start and end of the ramp (see Supplementary Table S1 for a comparison of these techniques). Dynamic calculations of assimilation, which include an accumulation term to account for changes in the concentration of CO_2_ in the chamber that is disregarded in steady-state equations, also make measurements of assimilation possible following sharp changes in [CO_2_]. With DATs, we can now use advanced ramps and step changes in [CO_2_] to clarify the mechanism by which TPU limitation causes oscillations, and how exactly the assimilation rate can surpass the steady-state limit.

## Materials and methods

### Plant materials and growth

*Nicotiana benthamiana* seeds were germinated in 2 liter pots of potting medium consisting of 70% peat moss, 21% perlite, and 9% vermiculite (Suremix; Michigan Grower Products Inc., Galesburg, MI, USA) in a greenhouse from June to August. This greenhouse was located at 42°43ʹN, 84°28ʹW, East Lansing, Michigan. Typical daylight light levels were between 300 µmol m^–2^ s^–1^ and 700 µmol m^–2^ s^–1^, and the daytime temperature was controlled to 27 °C. Plants were watered with half-strength Hoagland’s solution (Hoagland and Arnon, 1938) as needed as seedlings and then daily as adults. Plants were used for experiments from 6 to 7 weeks of age, and the uppermost fully expanded leaves were used for gas exchange.

### Combined optical measurements with gas exchange

A LI-COR 6800 12A 3 cm×3 cm clear top chamber (LI-COR Biosciences Inc., Lincoln, NE, USA) was connected to a scattering optic with an array of LEDs behind it (Hall *et al.*, 2013; Lantz *et al.*, 2019). The LI6800 12A backplate was replaced with a 3D-printed plate containing an optical and an infrared detector. The LED array contained actinic red and blue lights producing up to 2500 µmol m^–2^ s^–1^ with a ratio of 90% red (630 nm) and 10% blue (480 nm) light at 1000 µmol m^–2^ s^–1^. The saturation flash provided ~15 000 µmol m^–2^ s^–1^. Electrochromic shift (ECS) measurements were made with a 520 nm LED, with 505 nm used to correct for changes in zeaxanthin. The PSII operating efficiency (φ_II_) (Baker, 2008) was assessed by chlorophyll fluorescence using 520 nm as the excitation light. Measurements of PSI absorbance were made at 820 nm. While the absorbance at 820 nm may include other signals, such as reduced pheophytin or ferredoxin, these species are in low proportion and change more slowly than P700^+^ and should not significantly affect the kinetics (Christof and Ulrich, 1994). Measurements of PSI were taken according to Kanazawa *et al.* (2017) and measurements of ECS were taken according to Takizawa *et al.* (2007).

### Dynamic assimilation techniques

Dynamic measurements of gas exchange were made in a LI-COR 6800 with a LI-COR 6800 12A 3 cm×3 cm clear top chamber (LI-COR Biosciences Inc.). Plants were acclimated at experimental conditions until steady state with 1000 µmol m^–2^ s^–1^ photosynthetically active radiation and an air flow rate of 800 µmol s^–1^. Dynamic calibrations and range match were performed as recommended in the LI-COR 6800 version 2.0 manual (LI-COR, 2022). For experiments presented here, CO_2_ was ramped at rates of 100–500 ppm min^–1^ (~10–50 Pa CO_2_ min^–1^). Typical atmospheric pressure was 98 kPa.

### Dynamic assimilation with optical measurements

To take optical measurements along with the dynamic ramp of CO_2_, plants were first acclimated at 400 ppm CO_2_ and 1000 µmol m^–2^ s^–1^ light until steady state was achieved. CO_2_ was then abruptly lowered to 50 ppm CO_2_ at the reference IRGA, and the plant was acclimated at this CO_2_ level for 60 s. Afterwards, CO_2_ was ramped at a rate of 400 ppm min^–1^ (~40 Pa min^–1^) (or other rates as indicated) until 1500 ppm CO_2_ in the reference IRGA was recorded. (Because CO_2_ assimilation is a function of partial pressure, assimilation rates are reported as a function of partial pressure. However, the LI-COR 6800 mixes gases in terms of mole fraction, so in explaining experimental design, CO_2_ levels are given in mole fraction, ppm.) Typical atmospheric pressure at the site of experimentation was 98 kPa and was measured at the time of experimentation for exact calculations. A list of times from 20 s to 140 s in 10 s intervals was randomized, and individual ramps were performed sequentially for each interval, allowing assimilation to return to steady state at ambient CO_2_ before beginning the next ramp. At the chosen time, PSI and PSII activity, as well as the dark interval relaxation kinetics of the ECS, were measured.

## Results

### Oscillations are intensified when induced through ramps rather than CO _2_ step changes

**Fig. 1.**
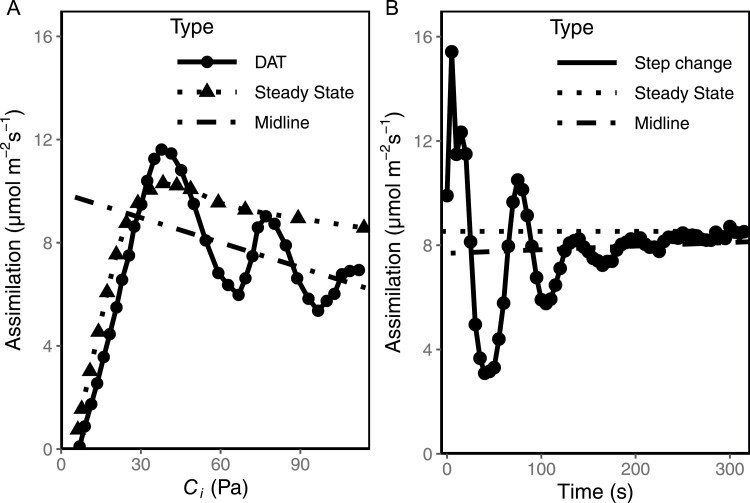
Oscillations induced by elevated CO_2_ compared with the steady state. (A) Full ramp of CO_2_ from 50 ppm to 1500 ppm at a rate of 400 ppm min^–1^ compared with a steady-state *A/C*_i_ curve (each measurement took 1–3 min for the steady-state method). (B) Oscillations induced by step change of CO_2_ from 50 ppm to 1400 ppm compared with the steady-state assimilation rate at 1400 ppm CO_2_. For both, a linear model is fit to the oscillating data to show the midline of oscillations.

The photosynthetic rate oscillated when the CO_2_ partial pressure was increased sufficiently to cause TPU limitation. When CO_2_ was ramped at 400 ppm min^–1^, oscillations were more pronounced than when CO_2_ was increased in a step change (Fig. 1). The higher amplitude/lower damping oscillations caused by a ramp up of CO_2_ resulted in a lower integral of *A* compared with an abrupt increase (Table 1). Oscillations induced by ramping CO_2_ resulted in, on average, a 20% loss of total assimilation compared with the steady state over the course of the ramp; significantly less at *P*=0.95. Oscillations induced by a step change of CO_2_ performed comparably to the steady-state assimilation value at the same CO_2_ level; no significant difference at *P*=0.95. We fitted a line through the middle of the oscillations. This midline trended down when oscillations were induced by a ramp of CO_2_ but trended up when CO_2_ was changed abruptly.

**Table 1.** A comparison of the total integrated assimilation during oscillations relative to the steady-state assimilation.

Type	Mean difference (%)	Difference SD	95% CI
Ramp	–20.0	2.6	–25.1 to –15.0
Step change	–2.2	3.9	–9.9 to 5.5

### Oscillations are induced specifically by entering TPU limitation

**Fig. 2.**
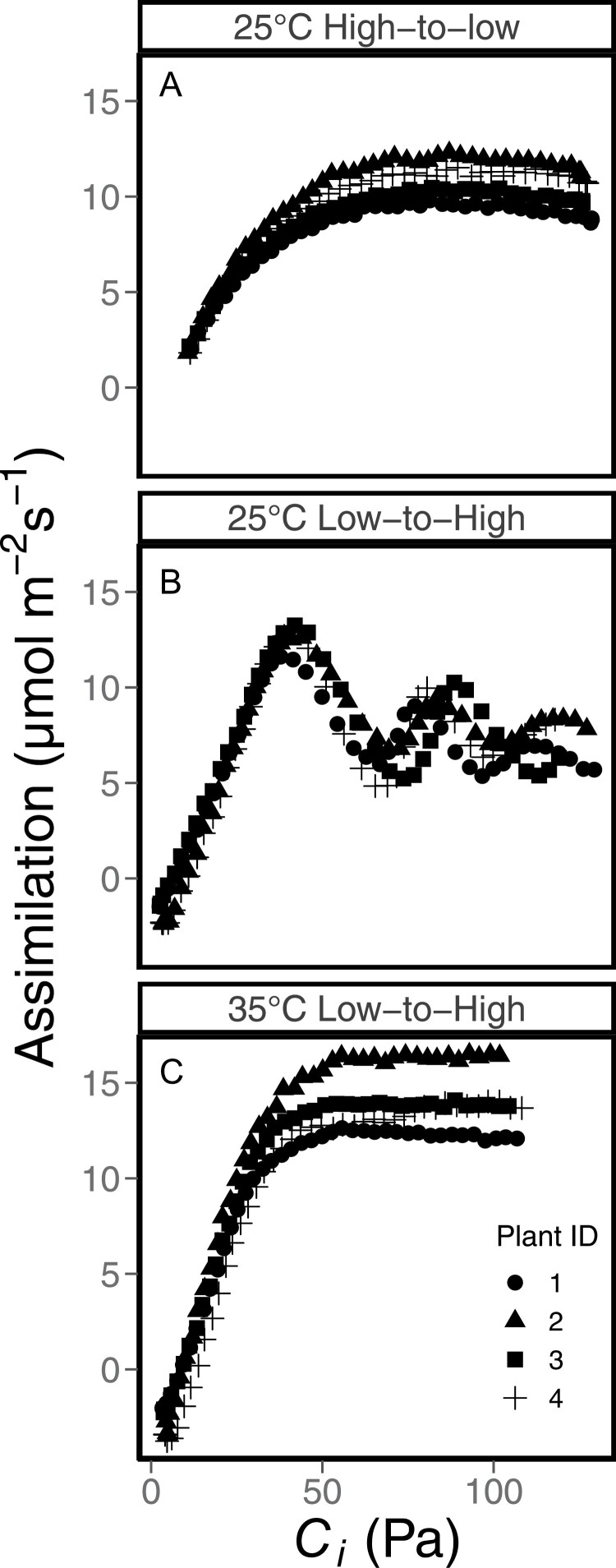
Assimilation measured using dynamic assimilation technique ramps of CO_2_ in three styles. (A) Reference CO_2_ is ramped from 1500 ppm to 50 ppm at 25 °C. (B) Reference CO_2_ is ramped from 50 ppm to 1500 ppm at 25 °C. (C) Reference CO_2_ is ramped from 50 ppm to 1500 ppm at 35 °C. For all curves, CO_2_ is ramped at a rate of 400 ppm min^–1^. Assimilation and *C*_i_ are logged every 5 s. Different symbols indicate replicate leaves.

Oscillations were observed only when plants entered TPU limitation (Fig. 2). Plants were acclimated at 400 ppm CO_2_ and either 25 °C (Fig. 2A, B) or, to prevent the occurrence of TPU limitation, 35 °C (Fig. C). Plants were then prepared to ramp through a range of CO_2_ values, starting at either 50 ppm (Fig. 2B, C) or 1500 ppm (Fig. 2A). Once the assimilation rates were steady, the CO_2_ was ramped through a range of CO_2_ values, either from 50 ppm to 1500 ppm (Fig. 2B, C) or from 1500 ppm to 50 ppm (Fig. 2A) at a rate of 400 ppm min^–1^. When measured at growth temperature and a ramp from low to high CO_2_, oscillations were observed beginning at a *C*_i_ of ~30 Pa. When ramped high to low, the plant did not exhibit oscillations at all. When ramped at a higher temperature to prevent TPU limitation from low to high, the plant did not exhibit oscillations. Therefore, the oscillations are caused specifically by entering TPU limitation, rather than any of the individual environmental ­conditions the plant experiences. Leaving TPU conditions does not result in oscillations.

### Oscillations are intensified when the ramp rate is increased

**Fig. 3.**
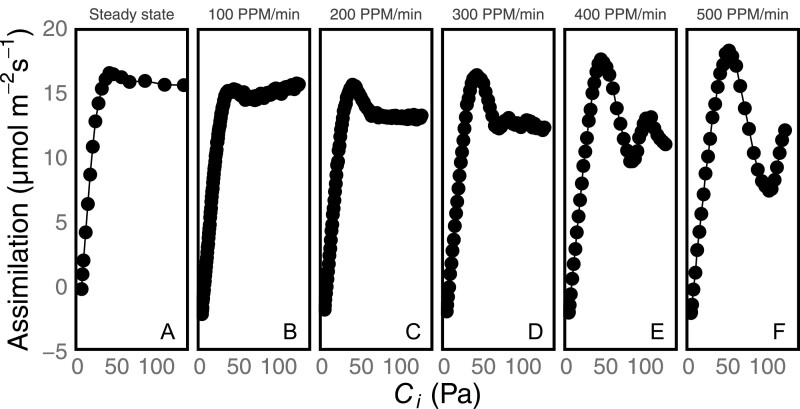
An example set of DAT ramps at various ramp rates, compared against the steady-state *A/C*_i_ curve. Reference CO_2_ is ramped from 50 ppm to 1500 ppm at rates of 100 (B), 200 (C), 300 (D), 400 (E), or 500 (F) ppm min^–1^ at 25 °C. For the steady-state *A/C*_i_ (A), 18 points were collected over a range of reference CO_2_ values from 50 ppm to 1500 ppm with 1–3 min between each point to allow for steady state to be reached. The amplitude of the oscillations increases in proportion to the ramp rate.

Plants were acclimated at ambient conditions then, after a 1 min delay at 50 ppm CO_2_, were ramped at a variable rate to 1500 ppm CO_2_ (Fig. 3). Sustained oscillations were not observed at a ramp rate of only 100 ppm CO_2_ min^–1^ but an initial peak was seen. The height of this first peak increased with ramp rate regardless of the appearance of oscillations (slope of peak versus ramp rate significant at *P*<0.05). The initial peak value of *A* was significantly greater at 300–500 ppm min^–1^ than at the steady-state rate (one-sided *t*-test, *P*<0.05). There was a corresponding increase in the depth of the following trough in assimilation rate as the ramp rate increased.

In Fig. 3, the assimilation rates are plotted versus *C*_i_. but there is also a time element given the variation in the rate of CO_2_ ramp. Supplementary Fig. S1 shows more examples of these same data, and Supplementary Fig. S2 shows the assimilation rates from Fig. 3 and Supplementary Fig. S1 but as a function of time (we put time on a log scale for convenience). Supplementary Fig. S2 shows that the peak assimilation rate decreases with time to reach said peak.

### Oscillations are intensified when TPU is enhanced through low temperature

**Fig. 4.**
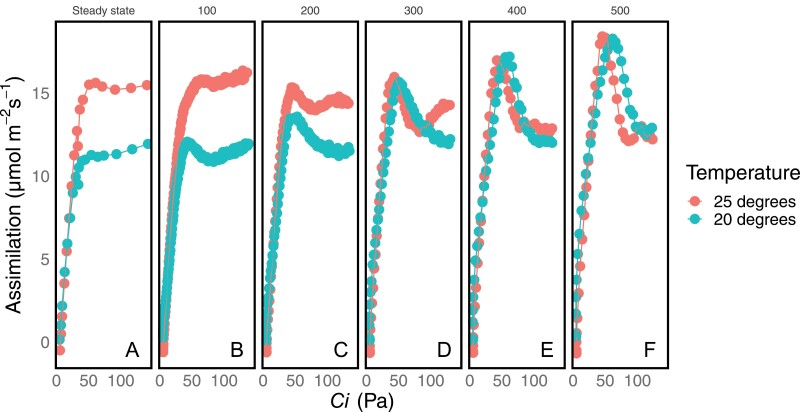
A set of DAT ramps at reduced temperature (20 °C) compared with ramps performed at growth temperature (25 °C). An 18 point steady-state *A/C*_i_ curve (A) is compared with DAT ramps in which reference CO_2_ is ramped from 50 ppm to 1500 ppm at rates of 100 (B), 200 (C), 300 (D), 400 (E), or 500 (F) ppm min^–1^. The amplitude of the induced oscillations at 20 °C increases with ramp rate, and is also greater than the amplitude of oscillations at 25 °C.

Plants were acclimated until steady state at 20 °C at 400 ppm CO_2_, then held at 50 ppm CO_2_ for 1 min before ramping from 50 ppm to 1500 ppm CO_2_ at a variable rate (Fig. 4). The peak amplitudes compared with the steady state were higher relative to those found at room temperature (*P*<0.1 by Welch’s *t*-test) when ramped at 300–500 ppm min^–1^. However, the absolute peak height is no different (*P*>0.1 by *t*-test) from the absolute peak height of ambient temperature ramps at 300–500 ppm min^–1^, despite being lower (*P*<0.1 by *t*-test) at 100–200 ppm min^–1^, as well as in the steady state. Additionally, the ramp rate required to achieve overshooting was lower, 200 ppm min^–1^ rather than 400 ppm min^–1^. These two components combined to increase the oscillation amplitude through the connecting factor of TPU capacity, even though they affect TPU limitation in different ways.

### Overshooting dynamically exceeds both TPU and the electron transport limitation of photosynthesis

**Fig. 5.**
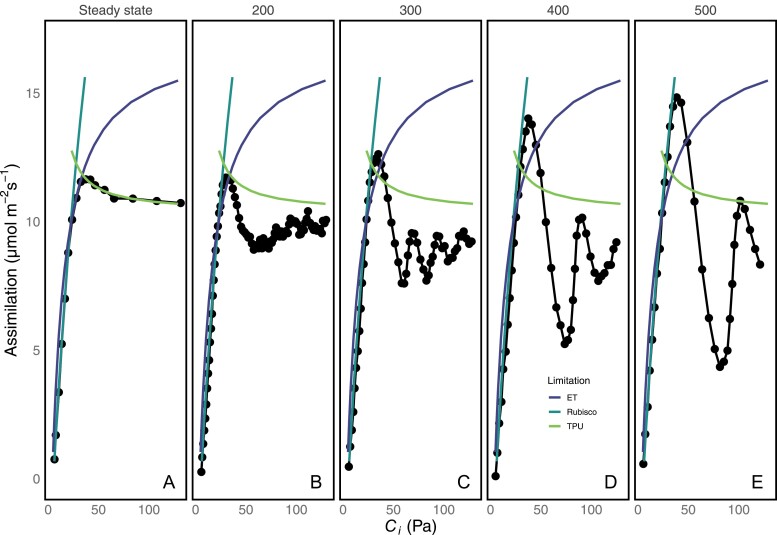
Comparison of oscillations versus fitting parameters from the steady-state *A/C*_i_. Fitting lines shown on each graph are determined solely from the steady-state *A/C*_i_ (A). Oscillations are induced by ramping from 50 ppm to 1500 ppm at rates of 200 (B), 300 (C), 400 (D), or 500 (E) ppm min^–1^. Oscillations can easily surpass TPU limitation, and at higher ramp rates can surpass the RuBP regeneration limitation but cannot surpass the rubisco limitation. At the highest ramp rates, the entire overshoot closely matches the rubisco limitation.

The oscillations caused by the CO_2_ ramp were plotted with limitations modeled from curve fitting (Gregory *et al.*, 2021) for data measured at discreet CO_2_ concentrations. Peak dynamic *A* often exceeded the steady-state TPU limitation during a ramp of CO_2_ (Fig. 5). At higher ramp rates, peak dynamic *A* also exceeded the RuBP regeneration limitation of photosynthesis. However, at no point did the overshoots exceed the rubisco limitation of photosynthesis.

### PSI reduction was involved in oscillations during CO _2_ ramps

**Fig. 6.**
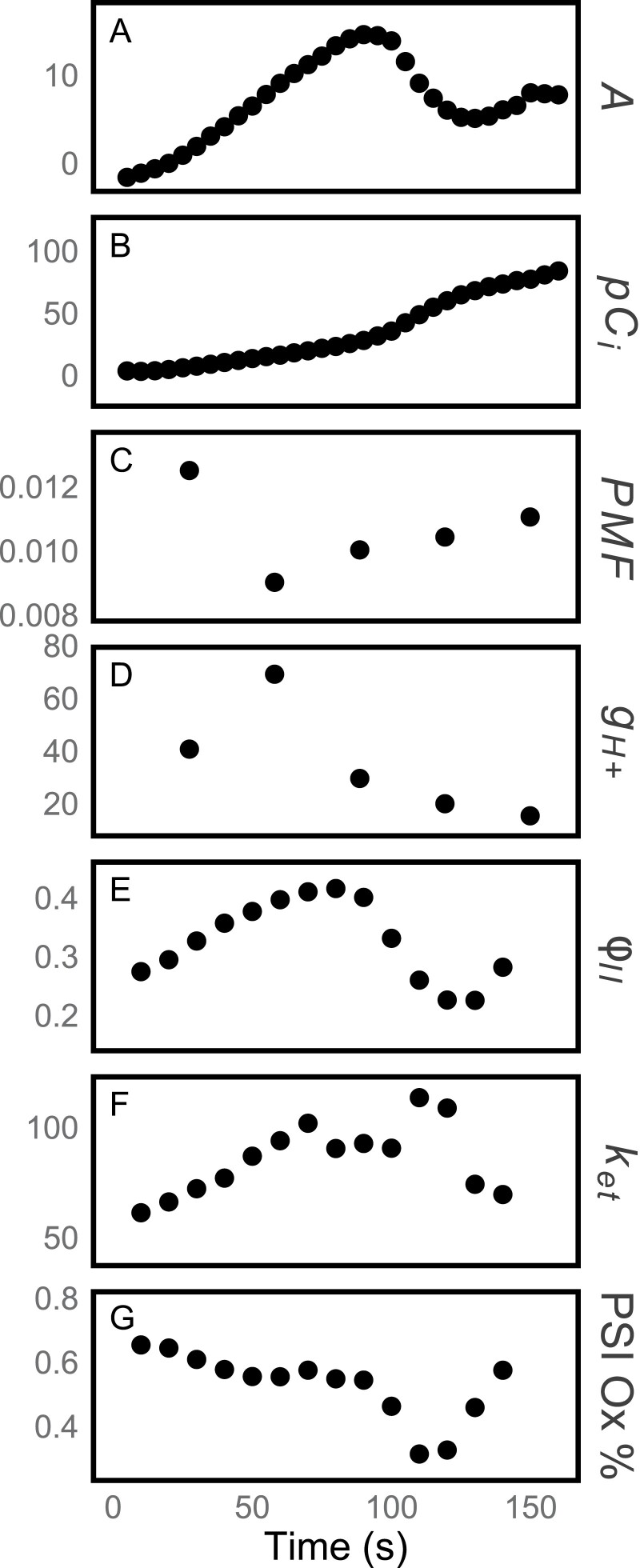
Combination of optical measurements with DAT. Oscillations in assimilation (A, in µmol m^–2^ s^–1^) are induced by ramping CO_2_ from 50 ppm to 1500 ppm at 400 ppm min^–1^. The increase in the internal partial pressure of CO_2_ (B, in Pa) increases non-linearly because it is affected by both the external CO_2_ and assimilation. φ_II_ (E) and PSI oxidation state (G) are calculated from saturation flashes. PMF (C), *g*_*H+*_ (D), and the rate of re-reduction of PSI by the cytochrome *b*_6_*f* complex (*k*_*et*_, F) are calculated from dark interval kinetics. *g*_*H+*_, φ_II_, and PSI oxidation state correspond to *A*, but PMF responds inversely to *A*.

Plants were ramped from 50 ppm to 1500 ppm CO_2_ in a special chamber adapted to house an LED array for measuring ECS and PSI oxidation in combination with PSII fluorescence (Fig. 6) based on components of the IdeaspeQ (Hall *et al.*, 2013). Assimilation and φ_II_ were correlated, as previously seen. However, PSI oxidation remained constant throughout the ramp until the first trough, at which point PSI oxidation fell (PSI became reduced). This suggests that the availability of NADP^+^ to accept electrons from PSI became limited.

## Discussion

Historically, most of the research on photosynthetic oscillations has been performed using sudden shifts in environmental conditions to induce oscillations. The use of ramps of varying speeds helps describe the phenomenology of oscillations to a greater degree, with some implications for the mechanisms of oscillations. The amplitude of oscillations resulting from ramps are greater and the oscillations damp more slowly than oscillations resulting from step changes (Table 2). Oscillations produced by step changes of CO_2_ tend towards the steady-state assimilation value. Oscillations produced by ramps, however, tend towards a different midline that diverges from the steady-state assimilation rate. We propose that this is due to the continuous change of the requirements for photosynthetic regulation, which is the damping force of these oscillations. The amplitude of the oscillations is also affected by the rate of the ramp. If the ramp is too slow, overshooting can still occur, but not oscillations. In this situation, a simple damped harmonic oscillator model cannot describe the behavior, as overshooting is not seen in an overdamped or critically damped model, and an underdamped model cannot account for the following trough.

**Table 2.** A comparison of the harmonic oscillator damping constants from a set of four plants, with each being tested in both oscillations induced by CO_2_ ramp and a step change in CO_2_

Replicate	Ramp damping ratio	Step change damping ratio
1	0.068	0.106
2	0.142	0.205
3	0.080	0.123
4	0.127	0.171

The damping constants were estimated by logarithmic descent of peak height. The mean difference is not 0 at *P*=0.95 using a two-sided paired *t*-test (95% CI 0.0291–0.065).

The use of ramps also allows us to compare the oscillations with the photosynthetic limitations fit from steady-state behavior. The peak exceeds the RuBP regeneration limitation and the TPU limitation, both of which are functions of metabolite pools. For short periods of time, metabolites such as RuBP can be used more rapidly than they are produced, depleting the pool and adding instability to the system. However, the rubisco limitation is not a function of metabolite pools; it is believed to represent the kinetics of RuBP-saturated rubisco and be unaffected by changes in RuBP pool size (Farquhar, 1979; Sharkey, 2023). It is therefore unsurprising that oscillations did not exceed the rubisco-limited portion of the curve. Because during transients the assimilation rate exceeds the RuBP and TPU limitations, oscillations prevent accurate measurement of *J* and TPU parameters associated with these limitations when they occur. Similar transient peaks in *A* above the steady-state rate of RuBP regeneration were induced by short periods of CO_2_-free air (Ruuska *et al.*, 1998). Short dark periods can also allow photosynthesis in subsequent light periods to exceed its steady-state rate for short periods (Stitt, 1986). On this basis, we propose that the overshooting achieved during oscillations results from the transient reduction in pools of metabolites which would otherwise be consumed at a steady rate, allowing photosynthesis to temporarily exceed the steady-state rate. In this model, the depth of the trough would be related to the quantity of newly produced metabolites from the peak that must be processed to restore metabolic balance. This is supported by the fact that oscillations did not result in overall more CO_2_ being fixed than in the steady state (Table 1). Because oscillations are induced by following a period of no TPU limitation with induction of TPU limitation, it is possible that the plant has plentiful inorganic phosphate free during the start of the ramp, and then the excess is used to transiently surpass the TPU limitation of photosynthesis. The subversion of the steady-state TPU-limited rate explains the similarity of ramps performed at 20 °C and 25 °C when the ramp speed is fast enough. TPU is the most temperature sensitive of the three components of photosynthesis as modeled here (McClain and Sharkey, 2019), so a subversion of TPU limitation brings the photosynthetic rate back in line with the photosynthetic rate achieved at 25 °C. Similarly, the plant should be able to dynamically exceed the RuBP regeneration-limited portion of the curve if RuBP is initially in excess. The height of the peak would then be related to the size of the available metabolite pool.

The occurrence of oscillations suggests the existence of an ‘acute’ TPU crisis that is rarely seen in the steady state. Reduction of PSI without a corresponding increase in electron flow from the cytochrome *b*_6_*f* complex means that availability of NADP^+^ must be limiting (Fig. 6). This situation could occur if there is insufficient ATP production to process 3-phosphoglyceric acid (PGA) into downstream products, limiting the flux through the reduction step. The troughs in assimilation that are lower than the steady state thus are caused by a lack of ATP, due to a sudden crisis in inorganic phosphate availability. This conclusion is supported by the decline in ATP synthase conductivity to protons (Fig. 6). This acute restriction shows the photosynthetic rate as limited by a rapidly changing TPU limitation in response to phosphate levels, as opposed to the steady state, which shows only the steady-state rate determined by the regulatory features that limit photosynthesis in response to TPU limitation. These data support the conclusions of Laisk *et al.* (1991), who also found reduction of P_700_ during oscillations and calculated that NADPH/NADP^+^ ratios were antiparallel with oscillations in both photosynthesis and in ATP/ADP ratios. In the stroma, phosphate must be at a lowered concentration to maximize sucrose (Huber and Huber, 1996) and starch synthesis (Preiss, 1982), but must remain at a sufficient concentration to drive ATP synthesis. In acute TPU limitation, the balance is disrupted by a short period of a very high photosynthetic rate. The transition from rubisco-limited to RuBP regeneration-limited conditions, and vice versa, involves much simpler adjustments in metabolism and so rarely produce oscillations.

The presence of an acute TPU crisis explains some non-obvious facets of steady-state TPU limitation. Triose phosphates do not necessarily build up in steady-state TPU limitation (Sharkey *et al.*, 1986b), a counterintuitive fact considering it is the first output of a cycle that, according to the model, is going too fast. Instead, it is common that RuBP builds up, which is unexpected as TPU limitation implicitly limits the ATP synthase and RuBP requires ATP to be regenerated. The lack of ATP causes PGA to increase by as much as 77% and RuBP pools shrink immediately after the imposition of TPU, but RuBP recovers as rubisco is deactivated (Sharkey *et al.*, 1986b) and presumably other regulatory mechanisms are engaged. It will take additional studies of the effect of transients in metabolite pools to examine these regulatory mechanisms.

The amplitude of the oscillations is affected by several factors. The plants will not begin oscillating unless they enter TPU limitation suddenly. Ramps that are too slow allow time for complex adjustments in metabolism and so do not induce oscillations, and the amplitude of the oscillations varies with the speed at which the plants are induced into TPU limitation. This is emphasized in Fig. 3, where the size of the overshoot varies with the length of time required to reach the beginning of oscillations. Plants ramped through an *A/C*_i_ curve at low temperature are particularly susceptible and will oscillate with greater amplitude. The greatest amplitude is seen in the initial overshoot and, if the initial peak does not overshoot, there are no oscillations seen (for instance, the 100 ppm min^–1^ and 200 ppm min^–1^ ramps in Fig. 3). If we believe that oscillations are caused by acute TPU limitation, the height of the overshoot will be related to the available metabolite pools usable before reaching a crisis in phosphate metabolism. When the ramp speed is fast, the integral of photosynthesis has been lower leading up to the beginning of oscillations, which would mean that the sum of metabolites consumed during the ramp is lower, while the potential to produce said metabolites should be approximately the same. When the plant reaches a *C*_i_ that would typically cause RuBP regeneration or TPU limitation, greater pool sizes would produce a higher peak.

If TPU limitation in the steady state is best described as a collection of regulatory components (McClain and Sharkey, 2019), these oscillations are the result of the time delays within those components. The strength of the perturbation is important to the phenomenology because it puts strain on photosynthetic regulation. Oscillations are damped over a period of a few minutes, enough time to activate PMF-dependent control through energy-dependent quenching and photosynthetic control at cytochrome *b*_6_*f*(Kramer and Crofts, 1993, 1996), as well as rubisco deactivation, which can begin in the first minutes of elevated CO_2_ (Sage *et al.*, 1988) or just 1 min of exposure to low O_2_ to induce TPU (Sharkey *et al.*, 1986b). Oscillations are seen when photosynthetic regulation is too slow to keep up with the changes in *A* and are damped when given enough time to activate regulatory controls on a time scale of minutes. This observation is supported by the reduced damping rate in oscillations induced via ramp (Table 2). The constantly changing setpoint for regulation causes the plant to perform less well and recover more slowly.

Speeding the response of photosynthesis-related processes has been seen as a method for increasing photosynthesis (Zhu *et al.*, 2004; Kromdijk *et al.*, 2016; Taylor and Long. 2017; Lawson and Vialet-Chabrand, 2019). It is possible that some of the mechanisms for increasing the speed of regulatory responses might trigger greater instabilities similar to those observed here as the rate of CO_2_ change was increased. It is possible that in a crop situation, the cost of the instabilities would not outweigh the advantage of faster responses; however, in nature over many generations, a more conservative approach to photosynthetic adaptations to stochasticity in the environment may have been favored.

### Conclusions

TPU limitation shows flexibility during dynamic assimilation measurements, for precisely the same reason it is insensitive to O_2_ and CO_2_ changes: it is separated from rubisco by layers of metabolites. In the steady state, inorganic phosphate pools are quite low (Sharkey and Vanderveer, 1989), but regulatory features balance the flux of inorganic phosphate into and out of the organic phosphate pool. Changing these fluxes dynamically imbalances photosynthesis and causes alternately a better and worse photosynthetic rate, and slower regulatory control is required to stabilize the photosynthetic rate again. This situation is a more intuitive understanding of TPU limitation—rather than being determined by a series of regulatory steps, the photosynthetic rate is determined by a crisis in metabolic pools.

At this point. it may be useful to divide the phenomenon of TPU limitation into two separate categories. In the steady state, TPU-limited photosynthesis is described primarily by regulatory features such as rubisco deactivation and reduced electron flow because of energy-dependent quenching. In the acute phase, however, the photosynthetic rate temporarily defies some assumptions of the three-limitation model of steady-state photosynthesis. Dynamic TPU limitation must be controlled by pool sizes, and it is reflected in electron transport dynamics.

## Supplementary data

Table S1. Comparison of the dynamic assimilation technique with other methods.

Fig. S1. Additional examples of oscillations induced by CO_2_ changes plotted against *C*_i_.

Fig. S2. Examples of oscillations in assimilation rate plotted against time instead of *C*_i_.

 Fig. S3. Five replicates of assimilation rates as affected by changing CO_2_ at different rates. Data obtained at 20 °C or 25 °C are shown.

erad084_suppl_Supplementary_Materials

## Data Availability

All data are available from the Dryad Depository doi.org/10.5061/dryad.n2z34tn10 (McClain and Sharkey, 2023).

## Abbreviations

*A*: CO_2_ assimilation rate
*C*_i_: intercellular CO_2_
DAT: dynamic assimilation technique
PMF: proton motive force
RACiR: rapid A/*C*_i_ response
TPU: triose phosphate utilization

## References

[CIT0001] Baker NR. 2008. Chlorophyll fluorescence: a probe of photosynthesis *in vivo*. Annual Review of Plant Biology 59, 89–113. 10.1146/annurev.arplant.59.032607.092759 18444897

[CIT0002] Cen Y , Sage RF. 2005. The regulation of Rubisco activity in response to variation in temperature and atmospheric CO_2_ partial pressure in sweet potato. Plant Physiology 139, 979–990. 16183840 10.1104/pp.105.066233 PMC1256011

[CIT0003] Christof K , Ulrich S. 1994. An improved method, using saturating light pulses, for the determination of photosystem I quantum yield via P700^+^-absorbance changes at 830 nm. Planta 192, 261–268.

[CIT0004] Cornic G , Louason G. 1980. The effects of O_2_ on net photosynthesis at low temperature (5°C). Plant, Cell & Environment 3, 149–157.

[CIT0005] Ellsworth DS , Crous KY, Lambers H, Cooke J. 2015. Phosphorus recycling in photorespiration maintains high photosynthetic capacity in woody species. Plant, Cell & Environment 38, 1142–1156. 10.1111/pce.12468 25311401

[CIT0006] Farquhar GD. 1979. Models describing the kinetics of ribulose biphosphate carboxylase-oxygenase. Archives of Biochemistry and Biophysics 193, 456–468. 464606 10.1016/0003-9861(79)90052-3

[CIT0007] Gregory LM , McClain AM, Kramer DM, Pardo JD, Smith KE, Tessmer OL, Walker BJ, Ziccardi LG, Sharkey TD. 2021. The triose phosphate utilization limitation of photosynthetic rate: out of global models but important for leaf models. Plant, Cell & Environment 44, 3223–3226. 10.1111/pce.14153 PMC9291784 34278582

[CIT0008] Guy CL , Huber JL, Huber SC. 1992. Sucrose phosphate synthase and sucrose accumulation at low temperature. Plant Physiology 100, 502–508. 16652990 10.1104/pp.100.1.502 PMC1075578

[CIT0009] Hall CC , Cruz JA, Zegarac R, DeMars D, Carpenter J, Kanazawa A, Kramer DM. 2013. Photosynthetic measurements with the Idea Spec: an integrated diode emitter array spectrophotometer/fluorometer. In: Photosynthesis research for food, fuel and the future. Berlin Heidelberg: Springer, 184–188.

[CIT0010] Harris GC , Cheesbrough JK, Walker DA. 1983. Effects of mannose on photosynthetic gas exchange in spinach. Plant Physiology 71, 108–111. 16662766 10.1104/pp.71.1.108 PMC1065994

[CIT0011] Hoagland DR , Arnon DI. 1938. The water culture method for growing plants without soil. California Agricricultural Experiment Station Circular 347. Berkley, CA.

[CIT0012] Holaday AS , Martindale W, Alred R, Brooks AL, Leegood RC. 1992. Changes in activities of enzymes of carbon metabolism in leaves during exposure of plants to low temperature. Plant Physiology 98, 1105–1114. 16668733 10.1104/pp.98.3.1105 PMC1080314

[CIT0013] Huber SC , Huber JL. 1996. Role and regulation of sucrose-phosphate synthase in higher plants. Annual Review of Plant Physiology and Plant Molecular Biology 47, 431–444. 10.1146/annurev.arplant.47.1.431 15012296

[CIT0014] Kanazawa A , Ostendorf E, Kohzuma K, et al. 2017. Chloroplast ATP synthase modulation of the thylakoid proton motive force: implications for photosystem I and photosystem II photoprotection. Frontiers in Plant Science 8, 719. 28515738 10.3389/fpls.2017.00719 PMC5413553

[CIT0015] Kiirats O , Cruz JA, Edwards GE, Kramer DM. 2009. Feedback limitation of photosynthesis at high CO_2_ acts by modulating the activity of the chloroplast ATP synthase. Functional Plant Biology 36, 893–901. 32688700 10.1071/FP09129

[CIT0016] Kramer DM , Crofts AR. 1993. The concerted reduction of the high- and low-potential chains of the bf complex by plastoquinol. Biochimica et Biophysica Acta 1183, 72–84.

[CIT0017] Kramer DM , Crofts AR. 1996. Control and measurement of photosynthetic electron transport in vivo. In: Baker NR, Govindjee, eds. Photosynthesis and the environment. Dordrecht: Kluwer Academic, 25–66

[CIT0018] Kromdijk J , Głowacka K, Leonelli L, Gabilly ST, Iwai M, Niyogi KK, Long SP. 2016. Improving photosynthesis and crop productivity by accelerating recovery from photoprotection. Science 354, 857–861. 27856901 10.1126/science.aai8878

[CIT0019] Labate CA , Leegood RC. 1988. Limitation of photosynthesis by changes in temperature: factors affecting the response of carbon-dioxide assimilation to temperature in barley leaves. Planta 173, 519–527. 24226689 10.1007/BF00958965

[CIT0020] Laisk A , Eichelmann H. 1989. Towards understanding oscillations: a mathematical model of the biochemistry of photosynthesis. Philosophical Transactions of the Royal Society B: Biological Sciences 323, 369–384.

[CIT0021] Laisk A , Eichelmann H, Oja V, Eatherall A, Walker DA. 1989. A mathematical model of the carbon metabolism in photosynthesis. Difficulties in explaining oscillations by fructose 2,6-bisphosphate regulation. Proceedings of the Royal Society B: Biological Sciences 237, 389–415.

[CIT0022] Laisk A , Siebke K, Gerst U, Eichelmann H, Oja V, Heber U. 1991. Oscillations in photosynthesis are initiated and supported by imbalances in the supply of ATP and NADPH to the Calvin cycle. Planta 185, 554–562. 24186534 10.1007/BF00202966

[CIT0023] Laisk A , Walker DA. 1986. Control of phosphate turnover as a rate-limiting factor and possible cause of oscillations in photosynthesis: a mathematical model. Proceedings of the Royal Society B: Biological Sciences 227, 281–302.

[CIT0024] Lantz AT , Solomon C, Gog L, McClain AM, Weraduwage SM, Cruz JA, Sharkey TD. 2019. Isoprene suppression by CO_2_ is not due to triose phosphate utilization (TPU) limitation. Frontiers in Forests and Global Change 2, 8.

[CIT0025] Laporte MM , Galagan JA, Prasch AL, Vanderveer PJ, Hanson DT, Shewmaker CK, Sharkey TD. 2001. Promoter strength and tissue specificity effects on growth of tomato plants transformed with maize sucrose-phosphate synthase. Planta 212, 817–822. 11346956 10.1007/s004250000433

[CIT0026] Lawson T , Vialet-Chabrand S. 2019. Speedy stomata, photosynthesis and plant water use efficiency. New Phytologist 221, 93–98. 29987878 10.1111/nph.15330

[CIT0027] Leegood RC , Edwards GE. 1996. Carbon metabolism and photorespiration: temperature dependence in relation to other environmental factors. In: Baker NR, ed. Photosynthesis and the environment. Dordrecht: Springer 191–221.

[CIT0028] LI-COR. 2022. Using the LI-6800 Portable Photosynthesis System. 9-66–9-109, licor.app.boxenterprise.net/s/kt6wwzmnvnlu4vc004pzp9u7cv9bvzj8.

[CIT0029] McClain AM , Sharkey TD. 2019. Triose phosphate utilization and beyond: from photosynthesis to end product synthesis. Journal of Experimental Botany 70, 1755–1766. 30868155 10.1093/jxb/erz058 PMC6939825

[CIT0030] McClain AM , Sharkey TD. 2023. Data from: Rapid CO_2_ changes cause oscillations in photosynthesis that implicate PSI acceptor-side limitations, under the original title ‘Triose phosphate utilization stress during photosynthesis addressed with dynamic assimilation measurements’. Dryad Digital Repository. 10.5061/dryad.n2z34tn10 PMC10199117 36883576

[CIT0031] Ogawa T. 1982. Simple oscillations in photosynthesis of higher plants. Biochimica et Biophysica Acta 681, 103–109.

[CIT0032] Peterson RB , Sivak MN, Walker DA. 1988. Carbon dioxide-induced oscillations in fluorescence and photosynthesis: role of thylakoid membrane energization in regulation of photosystem II activity. Plant Physiology 88, 1125–1130. 16666432 10.1104/pp.88.4.1125 PMC1055727

[CIT0033] Preiss J. 1982. Regulation of the biosynthesis and degradation of starch. Annual Review of Plant Physiology and Plant Molecular Biology 33, 431–454.

[CIT0034] Ruuska S , Andrews TJ, Badger MR, Hudson GS, Laisk A, Price DG, von Caemmerer S. 1998. The interplay between limiting processes in C_3_ photosynthesis studied by rapid-response gas exchange using transgenic tobacco impaired in photosynthesis. Australian Journal of Plant Physiology 25, 859–870.

[CIT0035] Saathoff AJ , Welles J. 2021. Gas exchange measurements in the unsteady state. Plant, Cell & Environment 44, 3509–3523. 10.1111/pce.14178 PMC9292621 34480484

[CIT0036] Sage RF , Kubien DS. 2007. The temperature response of C_3_ and C_4_ photosynthesis. Plant, Cell & Environment 30, 1086–1106. 10.1111/j.1365-3040.2007.01682.x 17661749

[CIT0037] Sage RF , Sharkey TD. 1987. The effect of temperature on the occurrence of O_2_ and CO_2_ insensitive photosynthesis in field grown plants. Plant Physiology 84, 658–664. 16665498 10.1104/pp.84.3.658 PMC1056646

[CIT0038] Sage RF , Sharkey TD, Seemann JR. 1988. The *in-vivo* response of the ribulose-1,5-bisphosphate carboxylase activation state and the pool sizes of photosynthetic metabolites to elevated CO_2_ in *Phaseolus vulgaris* L. Planta 174, 407–416. 24221524 10.1007/BF00959528

[CIT0039] Sharkey TD. 1985a. O_2_-insensitive photosynthesis in C_3_ plants. Plant Physiology 78, 71–75. 16664211 10.1104/pp.78.1.71 PMC1064678

[CIT0040] Sharkey TD. 1985b. Photosynthesis in intact leaves of C_3_ plants. The Botanical Review 5, 53–105.

[CIT0041] Sharkey TD. 2023. Maximising the efficiency of RuBP (ribulose bisphosphate) regeneration to optimise photosynthesis in crops. In: Sharwood R, ed. Understanding and improving crop photosynthesis. Cambridge: Burleigh Dodds Science Publishing Limited.

[CIT0042] Sharkey TD , Berry JA, Sage RF. 1988. Regulation of photosynthetic electron-transport in *Phaseolus vulgaris* L., as determined by room-temperature chlorophyll a fluorescence. Planta 176, 415–424. 24220871 10.1007/BF00395423

[CIT0043] Sharkey TD , Seemann JR, Berry JA. 1986a. Regulation of ribulose-1,5-bisphosphate carboxylase activity in response to changing partial pressure of O_2_ and light in *Phaseolus vulgaris*. Plant Physiology 81, 788–791. 16664903 10.1104/pp.81.3.788 PMC1075427

[CIT0044] Sharkey TD , Stitt M, Heineke D, Gerhardt R, Raschke K, Heldt HW. 1986b. Limitation of photosynthesis by carbon metabolism II. O_2_-insensitive CO_2_ uptake results from limitation of triose phosphate utilization. Plant Physiology 81, 1123–1129. 16664954 10.1104/pp.81.4.1123 PMC1075496

[CIT0045] Sharkey TD , Vanderveer PJ. 1989. Stromal phosphate concentration is low during feedback limited photosynthesis. Plant Physiology 91, 679–684. 16667087 10.1104/pp.91.2.679 PMC1062055

[CIT0046] Sivak MN , Walker DA. 1986. Photosynthesis *in vivo* can be limited by phosphate supply. New Phytologist 102, 499–512.

[CIT0047] Sivak MN , Walker DA. 1987. Oscillations and other symptoms of limitation of *in vivo* photosynthesis by inadequate phosphate supply to the chloroplast. Plant Physiology and Biochemistry 25, 635–648.

[CIT0048] Stinziano JR , McDermitt DK, Lynch DJ, Saathoff AJ, Morgan PB, Hanson DT. 2019. The rapid A/Ci response: a guide to best practices. New Phytologist 221, 625–627. 30198151 10.1111/nph.15383

[CIT0049] Stinziano JR , Morgan PB, Lynch DJ, Saathoff AJ, McDermitt DK, Hanson DT. 2017. The rapid A–Ci response: photosynthesis in the phenomic era. Plant, Cell & Environment 40, 1256–1262. 10.1111/pce.12911 28247953

[CIT0050] Stitt M. 1986. Limitation of photosynthesis by carbon metabolism I. Evidence for excess electron transport capacity in leaves carrying out photosynthesis in saturating light and CO_2_. Plant Phyisology 81, 1115–1122. 10.1104/pp.81.4.1115 PMC1075495 16664953

[CIT0051] Stitt M , Grosse H. 1988. Interactions between sucrose synthesis and CO_2_ fixation IV. Temperature-dependent adjustment of the relation between sucrose synthesis and CO_2_ fixation. Journal of Plant Physiology 133, 392–400.

[CIT0052] Stitt M , Kürzel B, Heldt HW. 1984. Control of photosynthetic sucrose synthesis by fructose 2,6-bisphosphate II. Partitioning between sucrose and starch. Plant Physiology 75, 554–560. 16663665 10.1104/pp.75.3.554 PMC1066954

[CIT0053] Takizawa K , Cruz JA, Kanazawa A, Kramer DM. 2007. The thylakoid proton motive force *in vivo*. Quantitative, non-invasive probes, energetics, and regulatory consequences of light-induced pmf. Biochimica et Biophysica Acta 1767, 1233–1244. 17765199 10.1016/j.bbabio.2007.07.006

[CIT0054] Takizawa K , Kanazawa A, Kramer DM. 2008. Depletion of stromal Pi induces high ‘energy-dependent’ antenna exciton quenching (qE) by decreasing proton conductivity at CFO-CF1 ATP synthase. Plant, Cell & Environment 31, 235–243. 10.1111/j.1365-3040.2007.01753.x 17996016

[CIT0055] Taylor SH , Long SP. 2017. Slow induction of photosynthesis on shade to sun transitions in wheat may cost at least 21% of productivity. Philosophical Transactions of the Royal Society B: Biological Sciences 372, 20160543. 10.1098/rstb.2016.0543 PMC5566890 28808109

[CIT0056] Walker DA , Sivak MN, Prinsley RT, Cheesbrough JK. 1983. Simultaneous measurement of oscillations in oxygen evolution and chlorophyll a fluorescence in leaf pieces. Plant Physiology 73, 542–549. 16663255 10.1104/pp.73.3.542 PMC1066503

[CIT0057] Yang JT , Preiser AL, Li Z, Weise SE, Sharkey TD. 2016. Triose phosphate use limitation of photosynthesis: short-term and long-term effects. Planta 243, 687–698. 26620947 10.1007/s00425-015-2436-8

[CIT0058] Zhu XG , Ort DR, Whitmarsh J, Long SP. 2004. The slow reversibility of photosystem II thermal energy dissipation on transfer from high to low light may cause large losses in carbon gain by crop canopies: a theoretical analysis. Journal of Experimental Botany 55, 1167–1175. 15133059 10.1093/jxb/erh141

