# Supplementary material for: Rapid CO2 changes cause oscillations in photosynthesis that implicate PSI acceptor-side limitations
Source: J Exp Bot. 2023 Mar 8;74(10):3163–73. doi: 10.1093/jxb/erad084 (PMC10199117; doi:10.1093/jxb/erad084)
Supplement: erad084_suppl_Supplementary_Materials [file erad084_suppl_supplementary_materials.pdf]

Supplemental information for

Short-term kinetics associated with triose phosphate utilization stress during photosynthesis addressed with dynamic assimilation measurements

Alan M. McClain and Thomas D. Sharkey

Correspondence: Thomas D. Sharkey, tsharkey@msu.edu

Containing

Table S1

Figures S1 – S3

	RACiR	Dynamic Assimilation Technique	Steady state
Pre-processing	Blank curve daily	User table configuration (once)	None
Matching	Single match before. None in the middle.	Range match once daily before running curves	Match every point
Post-processing	Blank fitting and subtraction; Ci adjustment	Nothing	Nothing
Speed	100 ppm/min recommended	Good up to 400 or 500 ppm/min	2-4 minutes per data point
Usable data range	Blank poor at the ends of the ramp	Generally good across the whole curve	Suitable for all reachable CO_2 values
Interface	Flexible but frustrating implementations	Well-behaved programs	Generally well-behaved
Data density	Log as frequently as you want		Comparatively very low
Fluorescence	Cannot be combined with fluorescence		Fluorescence capable
	Both are good for studying kinetics in assimilation!		Ignores all kinetics

Table S1: A comparison of Dynamic Assimilation Technique, RACiR, and steady state A/C_i techniques. We believe that DAT wholly supplants RACiR because of its ease of use, ease of data analysis, and better flexibility, especially with faster ramp rates. The high data density is its greatest asset when compared to steady-state A/C_i, but it doesn't combine with fluorescence and steady-state curves will be better in that regard.

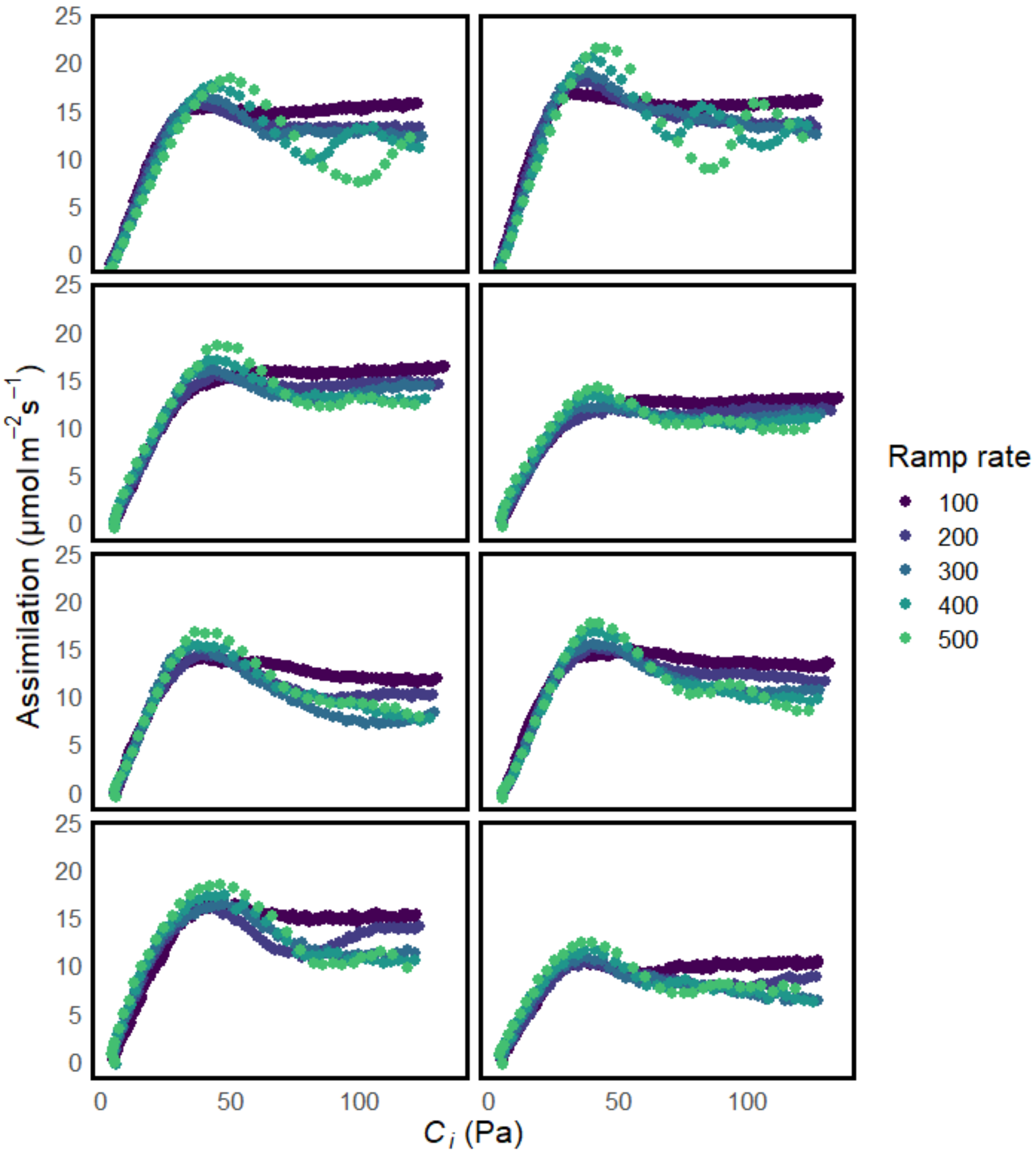

Figure S1: This figure complements figure 3 in the text. A set of DAT ramps from 50 to 1500 ppm reference CO_2 for 8 replicates, with ramp rates ranging from 100 ppm/min to 500 ppm/min, collected at 25°C. The amplitude of oscillations increases with ramp rate.

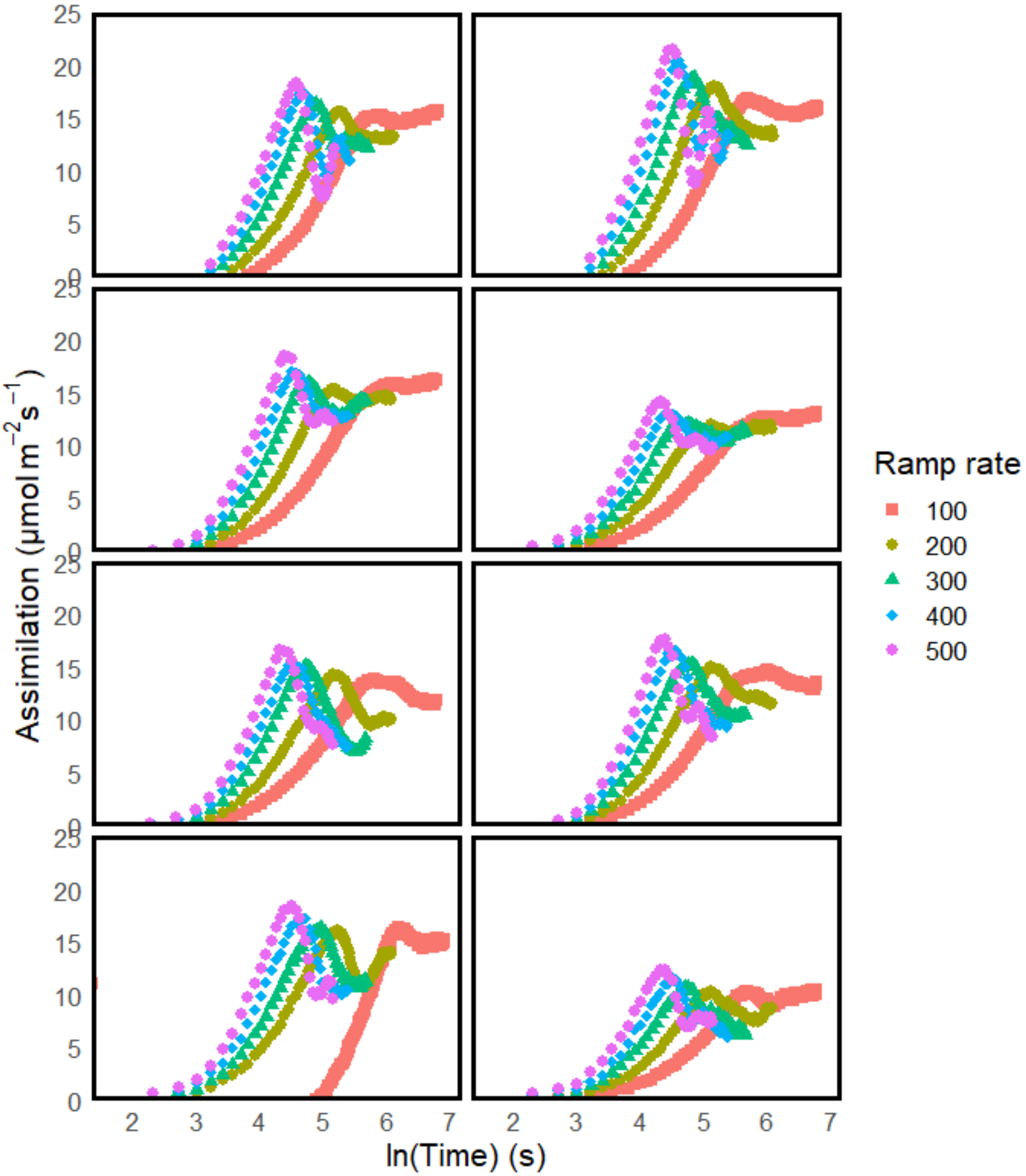

Figure S2: The same data from figure S1, but presented with log(Time) as the x-axis. Log transformation was performed as a visual aid. It is important to remember in these ramps that the increasing ramp rate also causes the plant to reach the apex of assimilation faster. It is probably not the increased ramp rate explicitly that really matters for achieving overshooting and oscillations, but the reduced time it takes to reach higher CO_2 values. As we see here, the greatest amplitude is seen with the highest ramp rate, and the least amount of time.

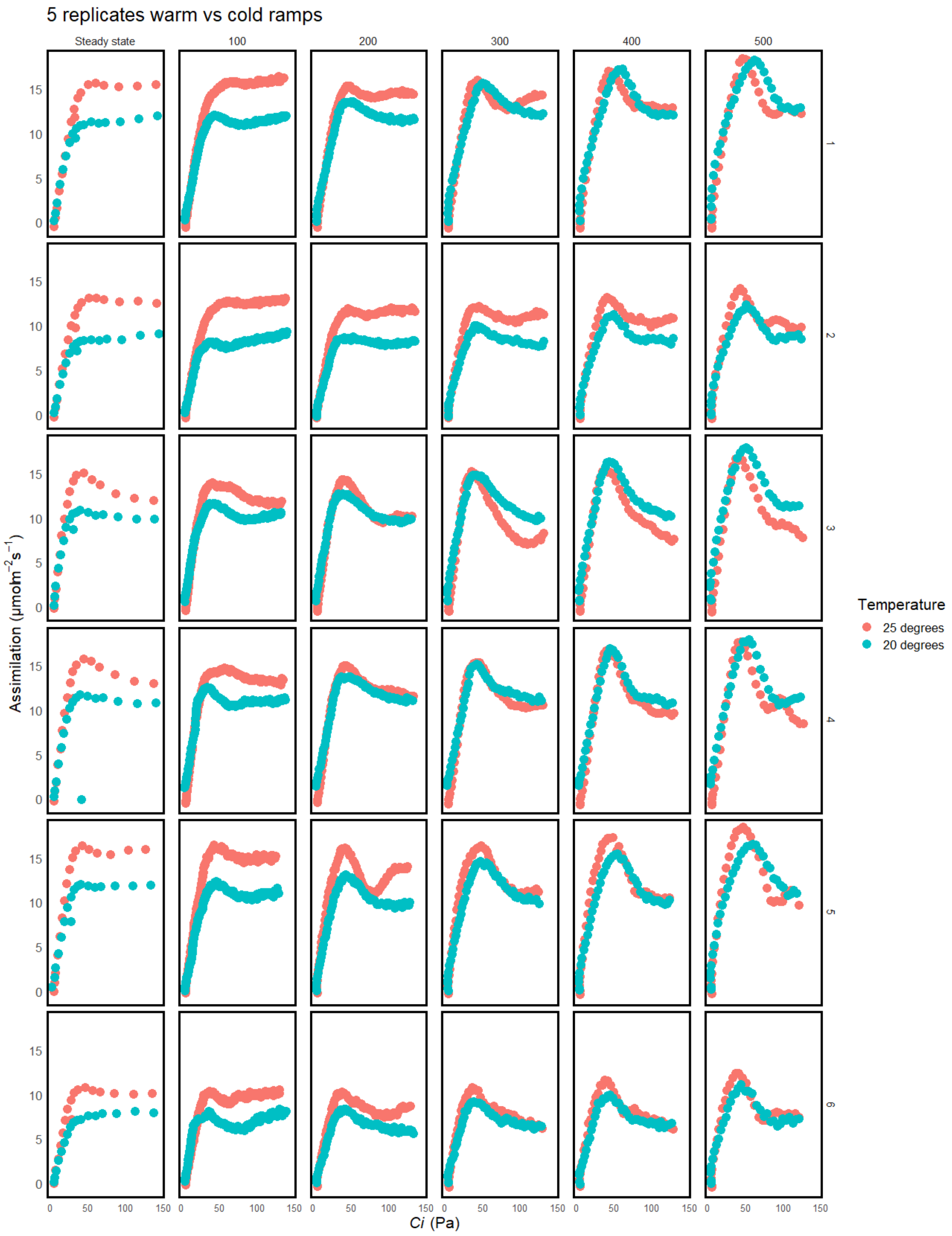

Figure S3: This supplemental figure accompanies Figure 4. These are the other replicates comparing overshooting and oscillations from ramps performed at ambient (25°C) or cool (20°C) temperatures. Each row is one replicate, and each column is a different ramp rate, from steady state to 100 ppm/min

to 500 ppm/min ramps. The assimilation rate is lower at the cool temperature than the ambient when the A/C_i is slow, but becomes more like the ambient rate at higher ramp rates.
